# Interventions to Increase the Rate of Influenza and Pneumococcal Vaccination in Patients with Chronic Obstructive Pulmonary Disease: A Scoping Review

**DOI:** 10.3390/medicina55060277

**Published:** 2019-06-15

**Authors:** Samuel P. Trethewey, Neil Patel, Alice M. Turner

**Affiliations:** 1 Respiratory Medicine, University Hospitals Birmingham NHS Foundation Trust, Birmingham B95SS, UK; s-trethewey@doctors.org.uk (S.P.T.); Neil.patel@heartofengland.nhs.uk (N.P.); 2 Institute of Applied Health Research, University of Birmingham, Birmingham B152TT, UK

**Keywords:** chronic obstructive pulmonary disease, COPD, vaccination, influenza, pneumococcal, interventions, adherence, compliance

## Abstract

*Background and Objective:* Current evidence suggests that patients with chronic obstructive pulmonary disease (COPD) should receive influenza and pneumococcal vaccinations. Despite international guidelines recommending vaccination in patients with COPD, many patients remain unvaccinated. Reasons for vaccine non-acceptance are multifaceted and are likely to be influenced by multiple psychosocial factors and pre-existing health beliefs. The aim of this review was to identify interventions which have been shown to effectively increase vaccination rates in patients with COPD. *Materials and Methods:* A structured search of PubMed returned 491 titles. Following title and abstract screening, seven full-text articles reporting on 6 unique interventional studies were extracted for narrative synthesis. A variety of interventions were investigated which, for the purposes of this review, were grouped into patient-focussed, clinician-focussed and mixed interventions. *Results:* Three papers reported findings from clinical trials (2 unique studies) and 4 papers reported findings from before-after studies. Two studies were conducted in the primary care setting, the remaining studies were conducted in secondary and tertiary care. Most studies reported both influenza and pneumococcal vaccination rates. These studies suggest that multimodal interventions, which target multiple aspects of evidence-based care and use both patient-focussed and clinician-focussed techniques, may have the greatest impact on vaccination rates in patients with COPD. *Conclusions:* Further, adequately powered, high quality studies are needed. It is crucial for individual institutions to monitor their own vaccination rates to determine if there is scope for performance improvement.

## 1. Introduction

Chronic obstructive pulmonary disease (COPD) is a progressive lung condition, characterised by partially reversible airflow obstruction, which is associated with symptoms including exertional breathlessness, chronic cough and regular sputum production [1]. Patients with COPD experience high morbidity and mortality, particularly in the context of acute exacerbations of COPD [2]. Patients with COPD have been reported to be at increased risk of developing influenza [3] and pneumococcal pneumonia [4] due to various mechanisms including impaired mucociliary clearance and increased production of specific cell adhesion molecules that mediate attachment of bacteria and viruses to airway epithelium [5,6,7].

In the UK, pneumonia accounts for more hospital admissions/bed-days than any other lung disease and is the third most common cause of death from lung disease [8]. Furthermore, pneumonia represents a significant cause of morbidity and mortality worldwide [9]. Analyses of data from the Global Burden of Disease Study demonstrated that *streptococcus pneumoniae* was the leading cause of lower respiratory infection morbidity and mortality globally in the year 2016 [10]. Moreover, the authors demonstrated that *streptococcus pneumoniae* contributed to more deaths (1,189,937 deaths) than all other lower respiratory infection aetiologies combined in 2016. Similarly, influenza has been shown to place a significant burden of morbidity and mortality on the global population, accounting for an estimated 9,459,000 hospitalisations and 145,000 deaths worldwide in the year 2017 [11]. 

### 1.1. Evidence for Vaccination in Patients with COPD

Current evidence suggests that COPD patients should be vaccinated against influenza and pneumococcal infections [12,13]. In a recent Cochrane systematic review update, Kopsaftis et al. [13] evaluated a total of six randomised controlled trials, comprising 2562 patients, which assessed the efficacy of influenza vaccination in patients with COPD or chronic bronchitis. The authors found that, compared with placebo, the inactivated influenza vaccine reduced the number of exacerbations in patients with COPD (mean difference –0.37 (95% CI, –0.64 to –0.11)). However, it is important to note that this was based on a limited number of trials (quantitative synthesis was only possible on two trials, comprising 180 patients) with varying degrees of risk of bias. Due to a lack of clinical equipoise, among other factors, it is not possible to conduct further placebo-controlled randomised controlled trials in specific populations including elderly patients with COPD [14]. Therefore, a large amount of evidence supporting influenza vaccination in patients with COPD has been derived from observational cohort studies. In a recent systematic literature review which, unlike the Cochrane review, included observational cohort studies, Bekkat-Berkani et al. [15] found that influenza vaccination reduced all-cause mortality and deaths associated with a respiratory event in patients with COPD. This finding was mainly driven by the UK-based study by Schembri et al. [16], which showed that influenza vaccination was associated with a 41% reduction in risk of all-cause mortality (RR 0.59 (95% CI, 0.57 to 0.61)).

The randomised controlled trial evidence supporting pneumococcal vaccination in patients with COPD is more robust. The most recent Cochrane systematic review of 12 randomised controlled trials, comprising 2171 patients, found moderate-quality evidence demonstrating a reduction in the rate of community-acquired pneumonia and COPD exacerbations in patients who received injectable polyvalent pneumococcal vaccination [12]. The number needed to treat (NNT) in order to prevent one episode of pneumonia was 19 (95% CI, 13 to 52) and the NNT to prevent one acute exacerbation of COPD was 8 (95% CI, 5 to 58) in the Cochrane systematic review.

### 1.2. Controversy and Barriers to Vaccine Acceptance

Despite the accumulating evidence in favour of vaccinating patients with COPD, and international guidelines recommending vaccination, a significant proportion of patients remain unvaccinated [17]. Reasons for this disparity are multifaceted and decisions to accept vaccination are likely to be influenced by multiple psychosocial factors and pre-existing health beliefs [18,19,20]. 

Controversy surrounding vaccination in patients with COPD is not a recent phenomenon [21]. As discussed in a leading respiratory journal commentary in 1987, “Enthusiasm for pneumococcal vaccines has waxed and waned for decades” [22]. Similarly, influenza vaccines have experienced their fair share of controversy [23]. The 2009 swine flu vaccine pandemic provided an interesting opportunity to explore reasons leading to the “anti-vaccine movement” concept [24]. An analysis of interviews with individuals who publicly criticised the pandemic flu vaccine identified several common motivators: concerns over safety due to a belief that the vaccine had been “rushed to market without proper investigation of its side effects”, a belief that “pharmaceutical companies had too much influence over this process”, a perception that the vaccine may contain other ingredients which may be associated with “serious diseases” and a perception that the pandemic influenza was not “a serious threat to most individuals’ health” meaning that the risks of vaccination would be greater than influenza infection [24]. Whether such concerns were valid is of course a matter for debate, and not within the scope of this article.

Using data comprising 36,811 patients with self-reported COPD in the US 2012 Behavioral Risk Factor Surveillance System (BRFSS), Hsu et al. [25] found the following factors to be negatively associated with influenza vaccination in patients with COPD: male gender, younger age, black or Hispanic ethnicity, lower education level, heavy alcohol use and current tobacco use. Of interest, a lack of access to a regular clinician was not identified as a factor associated with a lower likelihood of vaccination [25]. Similar barriers to pneumococcal vaccination have been observed in patients with COPD [26]. Many barriers are not clearly modifiable, such that improving vaccination rates remains a complex problem.

The importance of influenza and pneumococcal vaccination necessitates public health initiatives to increase vaccination rates in this patient population. This systematic literature review, therefore, aims to identify studies that have tested an intervention to increase vaccination rates in patients with COPD. This paper aims to provide a narrative synthesis of findings from these studies to identify interventions which may increase vaccination rates in patients with COPD. An additional aim of this review was to identify possible areas of further research. 

## 2. Methods

The PubMed database was searched from inception for studies investigating the effect of an intervention on influenza or pneumococcal vaccination rates in patients with a primary respiratory diagnosis of COPD. The following search terms were used: ((copd) AND ((vaccine) OR vaccination)) AND (((pneumococcal) OR pneumococcus) OR influenza). 

The search strategy returned a total of 491 papers. S.P.T. reviewed titles and abstracts to identify papers that described interventional studies in patients with COPD as per the primary aim of this review. Following title and abstract screening, seven full-text articles were extracted for narrative synthesis and summary results were reviewed by A.M.T. Table 1 summarises these articles.

## 3. Results

This scoping review identified a limited number of interventional studies, all of which were conducted over the past two decades, which report changes in vaccination rates in patients with COPD. It is important to note that, in these studies, increasing vaccination rates was not the primary aim. Rather, these studies comprised interventions, the majority of which were multimodal, which aimed to improve multiple aspects of care provided to patients with COPD. 

Three papers reported findings from clinical trials (two unique studies) and four papers reported findings from before-after studies. Two studies were conducted in a primary care setting. The remaining studies were conducted in secondary and tertiary care. Most studies reported both influenza and pneumococcal vaccination rates. For the purposes of this review, interventions have been grouped into: patient-focussed, clinician-focussed or a combination from both categories (mixed interventions). Table 2 gives a construct of the interventions evaluated in these studies.

### 3.1. Patient-Focussed Interventions

Two papers were identified that evaluated predominantly patient-focussed interventions [27,28]. These papers described the same study, but at different outcome points: 3-months and 12-months post-intervention, respectively. This study was a multicentre, non-randomised, quasi-experimental clinical trial investigating the impact of a bespoke patient education manual on the implementation of evidence-based practice in patients with moderate–severe COPD attending three hospitals in metropolitan Adelaide (South Australia). The rate of influenza vaccination was one of the primary outcomes of interest in this study.

The authors produced an 80-page, A5-sized patient education manual called “Talking to your doctor about COPD”, which contained 22 summaries of evidence on COPD treatments, predominantly based on Cochrane reviews. The manual was composed using best practice methods for presenting evidence to end-users and underwent several rounds of consultation and piloting with both patients and the wider multi-disciplinary healthcare team.

In total, 125 patients were recruited to the intervention group and 124 patients to the control group. Allocation was based on geographic region (patients in northern/western areas allocated to the control group and patients in southern areas allocated to the intervention group) to avoid patient-to-patient contamination via sharing of the manual between the groups.

There were no statistically significant differences between groups in rates of influenza vaccination at 3-months or at 12-months of follow-up. It is important to note that there was an unexpectedly high baseline rate of influenza vaccination in this study (87%–88%), which may have resulted in underpowering. The high baseline vaccination rates may have, in part, been due to the recruitment strategy. Of the 711 patients invited to take part in the study, only 249 agreed to take part, which could have resulted in significant selection bias. It is feasible that patients who agreed to take part in the study were more likely to engage with treatment recommendations generally and hence had a higher baseline vaccination rate. Nonetheless, despite extensive development and patient involvement, the authors failed to show a benefit of this comprehensive patient education manual with regards to the primary outcome of influenza vaccination rate in this cohort.

### 3.2. Clinician-Focussed Interventions

Four papers were identified that evaluated predominantly clinician-focussed interventions [29,30,31,33]. Morganroth et al. [29] conducted a multicentre, quasi-experimental, before-after study based in primary care investigating the impact of a performance improvement project in patients with COPD. Rates of influenza and pneumococcal vaccination were secondary outcomes in this study.

The authors recruited 242 COPD patients from 12 primary care clinics in the US. The intervention consisted of a bespoke, evidence-based, web-based, electronic resource for clinicians to guide their management of patients with COPD. The electronic resource was developed and piloted in partnership with pulmonologists and primary care physicians. The resource provided point-of-care support for various aspects of patient management including “(1) assessing and monitoring COPD, (2) reducing risk factors for COPD, (3) managing stable COPD and (4) managing acute COPD exacerbations.” In addition, the resource provided automated prompting regarding patient-specific care opportunities at the start of clinic visits/telephone consultations in addition to providing periodic audit and feedback data about individual physician performance.

This before-after analysis demonstrated an increased rate of pneumococcal vaccination (80% vs. 67%, *p* < 0.0001) but no difference in the rate of influenza vaccination (72% vs. 73%, *p* = 0.79) post-intervention. These findings suggest that a clinician-focussed web-based decision support tool may help to improve pneumococcal vaccination rates in this patient population. However, reasons for the lack of improvement in influenza vaccination rates post-intervention are unclear and warrant further evaluation.

Terasaki et al. [30] conducted a single-centre, before-after study, based in a tertiary care centre in the US, investigating the impact of an electronic COPD clinic flowsheet on patient care. A total of 411 patients were included in the analysis (144 pre-intervention and 267 post-intervention). The intervention consisted of an evidence-based, electronic COPD clinic flowsheet, which comprised a series of key questions regarding a patients’ clinical history. One of the goals of the flowsheet was to reduce documentation burden in the COPD clinic. Improving the rates of influenza and pneumococcal vaccination was not an explicit aim of this study, rather, the authors aimed to improve overall adherence to evidence-based care.

The authors demonstrated an increase in the rate of influenza vaccination (74% vs. 84%, *p* = 0.03) but no difference in the rate of pneumococcal vaccination (83% vs. 84%, *p* = 0.82) post-intervention. Interestingly, the flowsheet did not include a specific question regarding vaccination status. This suggests, therefore, that the improvement in influenza vaccination status either arose due to a general increase in clinician adherence to evidence-based practice or due to other confounding factors. The high baseline rate of pneumococcal vaccination may explain the lack of improvement in this outcome.

Zwar et al. [31] performed a multicentre, pragmatic, randomised controlled trial of the impact of a multimodal intervention consisting of nurse-general practitioner (GP) education, an electronic care-planning flowsheet and early intervention by the nurse-GP team. The study recruited a total of 254 patients with newly diagnosed COPD from 36 primary care practices (19 practices (144 patients) were randomised to the intervention group and 17 practices (110 patients) to the control group) in Sydney, Australia.

This study was interesting in that it excluded patients with a known diagnosis of COPD. Rather, the authors used a case-finding approach in which patients without known COPD were screened and were invited to attend a case-finding clinic appointment if they had risk factors for COPD (aged 40–85 years and with a documented history of smoking) and had attended the practice in the last 12 months. A total of 10,234 patients were invited. Of the 1641 patients who attended a case-finding appointment, 287 (18%) were diagnosed with COPD. The primary aim of this study was to determine whether the multimodal intervention improved quality-of-life (QoL) and process of care in this newly diagnosed COPD patient cohort. Influenza and pneumococcal vaccination rates were secondary outcomes in this study.

The authors observed an increased rate of influenza vaccination in the intervention group (73% vs. 57%, *p* = 0.035) but no difference in rate of pneumococcal vaccination between groups at 12-month follow-up. However, these findings should be taken in the context of an extremely low uptake of the intervention in this study. Only 22 patients (15.3%) in the intervention group saw the nurse for COPD care following case finding. The low intervention uptake places substantial limitations on the findings of this study. Moreover, the low response rate to the initial case-finding appointment invitations may have introduced significant bias into the study.

Burkes et al. [33] conducted a single-centre, before-after study on the impact of physician education and printed information cards for physicians on evidence-based care, including vaccination, in patients with COPD. Participants in this study comprised 570 COPD patients attending an internal medicine resident clinic (451 pre-intervention and 119 post-intervention) in the US. In contrast to other studies, this study placed more emphasis on vaccination as part of the intervention. The intervention consisted of physician education (a short PowerPoint-based session covering guideline-based COPD care and the plan for quality improvement) in addition to bespoke reference cards, which provided information regarding pneumococcal vaccination, the need for tobacco cessation counseling and diagnostic spirometry. Finally, the authors also implemented a standing order for pneumococcal vaccination, which enabled medical assistants to identify and vaccinate COPD patients in clinics.

The authors demonstrated an increased rate of pneumococcal vaccination (61% vs. 72%, *p* = 0.024) post-intervention. It is important to note that this positive result was achieved over a single cycle of quality improvement and, as the authors note, it remains to be determined if this improvement is sustainable in the long term. Notable strengths of this study include the simple, low-cost design of the interventions and central involvement of the internal medical residents.

### 3.3. Mixed Interventions

One paper was identified that evaluated a mixed intervention [32]. Bhatt et al. [32] conducted a single-centre, before-after study of 187 COPD patients (109 pre-intervention, 78 post-intervention) admitted with an acute exacerbation in Alabama, US. The authors aimed to determine if a multimodal intervention could reduce 30-day all-cause readmission rates for COPD exacerbations and reduce overall costs. Vaccination rates were exploratory outcomes in this study.

The intervention comprised patient education material (including details of red-flag symptoms), rapid review in a COPD clinic post-acute exacerbation (within two weeks) and periodic follow-up phone calls from a practice nurse.

There were no differences in either of the primary outcomes in this study. However, the authors observed significantly increased rates of influenza vaccination (31% vs. 51%, *p* = 0.011) and pneumococcal vaccination (37% vs. 82%, *p* < 0.001) post-intervention. Despite being limited by a small sample size, the findings from this study suggest that an acute admission, due to an exacerbation of COPD, can provide an opportunity for greater patient engagement with evidence-based care including vaccination. Furthermore, this study suggests that multimodal interventions, which combine patient and clinician-focussed interventions, may have the greatest effect on vaccination rates.

## 4. Discussion

This scoping review identified seven papers, reporting on six unique interventional studies that evaluated influenza and pneumococcal vaccination rates in patients with COPD. It is important to note that none of these studies aimed to improve vaccination rates alone, rather they sought to improve multiple aspects of evidence-based care in patients with COPD. Moreover, vaccination was often a secondary or exploratory outcome in these studies. Consequently, conclusions must be made with caution.

Baseline rates of influenza and pneumococcal vaccination varied greatly between studies, reflecting heterogeneity of study design, including geographical location, specific populations targeted (primary vs. secondary care) and recruitment strategies. In some cases, the baseline rate of vaccination was high, resulting in underpowered studies. As previously discussed, the high baseline vaccination rate seen in some of the studies may have resulted from selection bias due to study methodology.

We grouped interventions broadly into patient-focussed, clinician-focussed and mixed interventions. The main patient-focussed intervention evaluated in these studies involved patient education regarding evidence-based care. Vaccination is a topic fraught with controversy, which is often a result of the spread of misinformation and misunderstanding [34]. Patient education, therefore, appears to be a sensible method to increase patient understanding of the evidence base for vaccination in COPD. One way to supplement patient understanding of specific vaccines is to provide additional educational material, as was performed in the studies by Harris et al. [27,28] and Bhatt et al. [32]. Although the study by Harris et al. [27,28] did not show a significant difference in rate of vaccination, several important limitations may have influenced this finding, as previously discussed. It is also possible that the negative results observed in the study by Harris et al. [27,28] may have resulted from an approach that only targeted one aspect of care—patient education. Other studies, based in the acute care setting, have shown that interventions that consist of education alone do not result in significant increases in influenza and pneumococcal vaccination rates [35]. The positive results observed in the before-after study by Bhatt et al. [32] highlights the importance of using a multimodal approach to improve the rate of influenza and pneumococcal vaccination in patients with COPD. Due to the multiple factors relating to vaccine acceptance, it is unlikely that a single intervention will result in a dramatic increase in vaccination rate.

It is plausible that certain health behaviours are closely related. For example, it is perhaps unsurprising that patients who continue to smoke and who report heavy alcohol use are observed to have lower adherence to recommended vaccinations [25]. Negative health behaviours may cluster together in this way, such that patients who engage in one negative health behaviour may be more likely to engage in another negative health behaviour [36]. To optimise the care of patients with COPD and to increase vaccination rates, it may therefore be more efficacious to take a holistic approach and target multiple aspects of care simultaneously. For example, smoking cessation advice should go together with vaccine recommendations and with other aspects of evidence-based care.

Based on findings from the studies included in this review, there is an argument for using clinician-focussed decision support tools, including flowsheets and checklists, to improve aspects of evidence-based care in patients with COPD. Simple decision support tools may be easier to implement, have a shorter learning curve and may be more likely to be used in the context of a busy respiratory or primary care clinic. The obvious benefit of decision support tools is that they act as a reminder to busy clinicians regarding important aspects of care that need to be reviewed during a clinic visit. For example, in acute COPD admissions care bundles (which are effectively a checklist of actions to perform when caring for a patient) have been used to good effect in both improving use of evidence-based care and subsequent outcomes [37].

Trust is a key determinant of patient behaviour [38]. Patients who trust their physician may be more likely to adhere to treatment recommendations [39]. It is therefore crucial that this is not overlooked during consultations in which vaccination is discussed with patients. It is important to explore patient understanding regarding specific vaccinations and address any concerns they might have. Recent research has shown that using a narrative medicine (patient-centred) approach, which focusses on cultivating “positive patient-doctor relationships based on listening and understanding”, can improve adherence to treatment recommendations in patients with COPD [40].

Other possible interventions, not evaluated in the studies identified by this review, include greater media/community engagement, regular monitoring of local vaccination rates and greater involvement of non-prescribing healthcare professionals in the screening, counselling and administration of vaccines [41]. Regular monitoring of local vaccination rates may encourage quality improvement and greater involvement of the wider multidisciplinary team may improve vaccination rates in patients with COPD, however this requires further research [42].

## 5. Conclusions

Methodological heterogeneity and modest sample sizes of included studies make it difficult to draw definitive conclusions regarding the most optimal interventions to increase influenza and pneumococcal vaccination rates in patients with COPD. Further, adequately powered, high quality studies are needed. A focus of research should be on multimodal approaches, combining patient- and clinician-focussed interventions.

Finally, it is crucial for individual centres to monitor their own vaccination rates to determine if there is room for improvement, before embarking on ambitious performance improvement projects. Key stakeholders need to determine if directing time and resources towards projects to increase vaccination rates is a worthwhile objective, as high baseline vaccination rates may hinder performance improvement.

## Figures and Tables

**Table 1:** Studies of interventions to increase vaccination rates in patients with COPD.

Study	Design	Intervention	Findings
Harris 2006 [27]	Moderate-severe COPD in secondary care; multicentre, quasi-experimental clinical trial; *n* = 249	Evidence-based patient manual	No difference in rate of influenza vaccination between intervention and control groups at 3 months follow-up
Harris 2009 [28]	Moderate-severe COPD in secondary care; multicentre, quasi-experimental clinical trial; *n* = 249	Evidence-based patient manual	No difference in rate of influenza vaccination between intervention and control groups at 12 months follow-up
Morganroth 2015 [29]	COPD in primary care; multicentre, quasi-experimental, before-after study; *n* = 242	Evidence-based, electronic, COPD decision support tool	Increased rate of pneumococcal vaccination (67% to 80%, *p* < 0.0001) but no difference in rate of influenza vaccination post-intervention
Terasaki 2015 [30]	Stable COPD in tertiary care; single-centre, before-after study; *n* = 411	Evidence-based, electronic, COPD clinic flowsheet	Increased rate of influenza vaccination (74% to 84%, *p* = 0.03) but no difference in rate of pneumococcal vaccination post-intervention
Zwar 2016 [31]	Newly diagnosed COPD in primary care; multicentre, pragmatic randomized controlled trial; *n* = 254	Nurse-GP education; electronic care-planning flowsheet; early intervention by the nurse-GP team	Increased rate of influenza vaccination (73% versus 57%, *p* = 0.035) in intervention group but no difference in rate of pneumococcal vaccination between intervention and control groups
Bhatt 2017 [32]	Post-acute exacerbation COPD in secondary care; single-centre, before-after study; *n* = 187	Patient education material; rapid review in COPD clinic post-exacerbation; periodic follow-up phone calls from a nurse	Increased rates of influenza vaccination (31% to 51%, *p* = 0.011) and pneumococcal vaccination (37% to 82%, *p* < 0.001) post-intervention
Burkes 2018 [33]	COPD in secondary care; single-centre, before-after study; *n* = 570	Physician education, printed evidence-based reference cards for physicians; standing order of vaccines	Increased rate of pneumococcal vaccination (61% to 72%, *p* = 0.024) post-intervention

**Table 2:** Overview of types of interventions.

Patient-Focussed	Clinician-Focussed
Patient education Face-to-face Written information	Clinician education Face-to-face E-learning Written information
Early intervention Newly-diagnosed COPD Post-acute exacerbation	Decision support tools (electronic or paper) Flowsheets Templates/checklists Prompts/reminders Reference information

Mixed interventions contain elements from both patient- and clinician focused.
